# Chronic HCV Infection Is Associated with Overexpression of Human Endogenous Retroviruses that Persists after Drug-Induced Viral Clearance

**DOI:** 10.3390/ijms21113980

**Published:** 2020-06-01

**Authors:** Pier-Angelo Tovo, Silvia Garazzino, Valentina Daprà, Carla Alliaudi, Erika Silvestro, Cristina Calvi, Paola Montanari, Ilaria Galliano, Massimiliano Bergallo

**Affiliations:** 1 Department of Pediatric Sciences and Public Health, University of Turin, Piazza Polonia 94, 10126 Turin, Italy; carla.alliaudi@unito.it (C.A.); cristina.calvi@unito.it (C.C.); paola.montanari@unito.it (P.M.); ilaria.galliano@unito.it (I.G.); 2 Department of Pediatrics, Infectious Diseases Unit, Regina Margherita Children’s Hospital, Piazza Polonia 94, 10126 Turin, Italy; silvia.garazzino@unito.it (S.G.); erika.silvestro@unito.it (E.S.); 3 Pediatric Laboratory, Department of Pediatric Sciences and Public Health, University of Turin, 10126 Turin, Italy; valentina.dapr@yahoo.it

**Keywords:** hepatitis C virus infection, human endogenous retroviruses, viral clearance, cancers, autoimmune diseases

## Abstract

Chronic hepatitis C virus (HCV) infection is associated with several hepatic and extrahepatic complications, including cancers and autoimmune disorders, whose frequency is reduced but not abolished after drug-induced viral clearance. The causes of these complications and of their persistence are ill-defined. Human endogenous retroviruses (HERVs) are remnants of ancestral infections and constitute 8% of the human genome. Most HERV elements are inactive, but some are transcribed. HERV overexpression is associated with many cancers and autoimmune diseases with a putative pathogenetic role. Several viral infections trigger HERV activation, but there are no studies on HCV-infected subjects. We assessed, through a PCR real-time amplification assay, the transcription levels of the pol genes of HERV-H, -K, and -W, and of their repressor TRIM28 in white blood cells (WBCs) of vertically infected children, both before and after therapy with direct-acting antivirals (DAAs). The results documented significantly higher expressions of HERV-H-pol and HERV-K-pol, not of HERV-W-pol, in HCV-infected subjects as compared to age-matched controls. HERV RNA levels remained unchanged after DAA-driven viral clearance. No significant variations in transcription levels of TRIM28 were observed in infected subjects. Our findings demonstrate HERV-H-pol and HERV-K-pol overexpression in subjects with chronic HCV infection, without variations after a positive response to DAAs; this might justify their predisposition to cancers and autoimmune disorders that persist after a DAA-induced resolution of viremia.

## 1. Introduction

Chronic hepatitis C virus (HCV) infection is associated with a large array of hepatic and extrahepatic complications [1]. These include a number of malignancies, such as hepatocellular carcinoma (HCC), B cell lymphoma [2], and other solid tumors [3,4], as well as autoimmune manifestations, such as cryoglobulins, membranoproliferative glomerulonephritis, and thyroid diseases [5]. Eradication of infection, either with interferon alfa or with direct-acting antivirals (DAAs), has reduced but not abolished the predisposition to cancers [6,7]. Even the risk of autoimmune disorders does not disappear after the resolution of viremia. In our cohort of vertically HCV-infected children, cryoglobulins and non-organ-specific autoantibodies persisted or developed in subjects with spontaneous viral clearance [8,9]. Meanwhile, it became clear that the persistence or appearance of autoantibodies and cryoglobulins, as well as relapses of vascular disorders, may occur in a significant proportion of adults with a positive response to direct-acting antivirals (DAAs) [10,11]. The cause of the predisposition to cancers and autoimmune diseases in HCV-infected subjects, and of its persistence after spontaneous or drug-induced viral clearance, remains an unsolved dilemma.

Human endogenous retroviruses (HERVs) constitute about 8% of the human genome. They are the remnants of ancestral infections that led to their integration into the DNA of primates, more than 25 million years ago [12]. During evolution, the accumulation of mutations, deletions, and recombinations blocked the production of infectious virions. Most HERV elements have become inactive through progressive mutations or epigenetic silencing. However, some are transcribed, and a few encode viral proteins, such as the syncytins that are involved in placenta morphogenesis and in feto-maternal tolerance, but postnatally may favor cancer development and diffusion [13,14]. HERVs can regulate the transcription of adjacent cellular genes and generate novel insertions into the genome by retrotransposition of their pseudogenes [12]. Aberrant HERV expressions have been found in association with a number of cancers [15,16] and autoimmune disorders [17,18] supporting their putative pathogenetic role in these pathologies. Among epigenetic factors that can regulate HERV expression, the host factor tripartite motif-containing-28 (TRIM28, also known as KAP1 or TIF1β) is a transcriptional corepressor acting in concert with Krüppel-associated box domain-zinc finger proteins (KRAB-ZNFs) to induce DNA methylation and silencing of endogenous retroviral elements [19,20].

Several exogenous viral infections can trigger HERV activation [21,22,23,24] and, recently, wild-type influenza A virus has been shown to induce alterations of TRIM28 that reduce the repression of endogenous retroviruses [25]. HERV activation has not been explored in chronic HCV infection, either before or after the resolution of viremia. Several groups of HERVs have been identified; among these, HERV-H, HERV-K, and HERV-W are those most widely studied. The aims of this study were to assess the transcription levels of the pol genes of HERV-H, -K, and -W in white blood cells (WBCs) of HCV RNA+ children and adolescents with vertically acquired infection, their variations following DAA-induced viral clearance, and to determine whether abnormal HERV transcriptions were associated with alterations of their repressor TRIM28.

## 2. Results

### 2.1. Study Populations

Seventeen HCV RNA+ subjects were studied (6 males, 11 females; median age 12.49 years, range 1.23–17.37). Their viral genotypes were type 1 in 14 cases, type 2 in 1 case, and type 4 in 2 cases. General condition was good in all patients, and none had other pathologies, including autoimmune diseases. Four had mild biochemical signs of hepatitis (ALT mean values +/− SD: 83 U/mL +/− 31.4); four had cryoglobulinemia, one patient had ANA, and one had anti-LKM-1 antibodies. No cases of cirrhosis were detected.

The control population included 37 age-matched control subjects (24 males, 13 females; median age 12.39 years, range 2.05–16.60).

### 2.2. Expression Levels of Housekeeping Gene GAPDH

Expression of housekeeping gene GAPDH was similar between HCV-infected subjects and age-matched controls (mean +/− SD: 21.83 +/− 0.38 ct vs. 21.81 +/− 0.73 ct, respectively, *p* = 0.6474).

### 2.3. Transcription Levels of HERV-H-pol, HERV-K-pol, and HERV-W-pol in HCV RNA+ Subjects and Control Group

**Figure 1:**
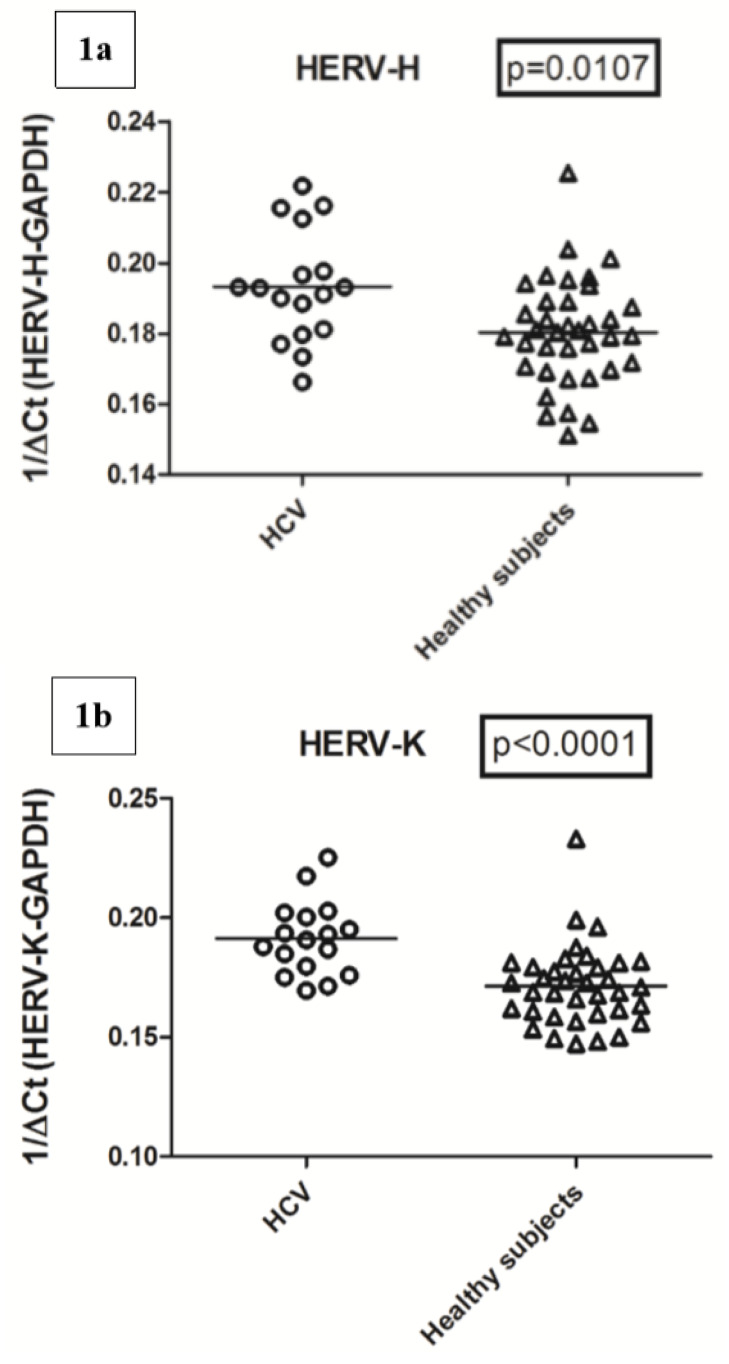

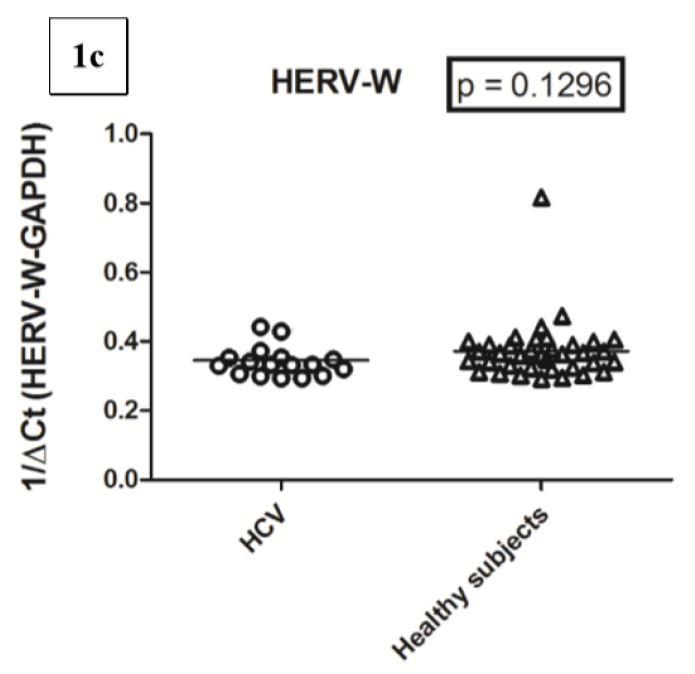
Transcriptional levels of the pol genes of human endogenous retroviruses (HERVs), HERV-H (**a**), HERV-K (**b**), and HERV-W (**c**) in white blood cells (WBCs) from hepatitis C virus (HCV) vertically infected subjects and age-matched control subjects. Circles and triangles show transcriptional levels of each subject; these are represented by 1/ΔCt. Statistical analysis through the Mann–Whitney test.

The transcription levels of the pol genes of HERV-H and HERV-K were significantly higher in HCV-infected patients than in the age-matched control subjects, whereas those of HERV-W did not show a significant difference (Figure 1).

In particular, the HERV-H-pol values (mean +/− SD) were 0.192 +/− 0.015 in HCV+ subjects vs. 0.180 +/− 0.015 in control subjects; the HERV-K-pol values were 0.190 +/− 0.016 in HCV+ vs. 0.171 +/− 0.017 in control subjects. The transcription levels of HERV-W-pol values were 0.346 +/− 0.049 in HCV+ vs. 0.372 +/− 0.086 in controls.

### 2.4. Transcription Levels of HERV-H-pol, HERV-K-pol, and HERV-W-pol Following Therapy with DAAs

All the eight HCV-infected subjects (median age 13.10 years, range 12.42–17.07; 5 males, 3 females; 6 genotype 1, 2 genotype 4) who were treated with sofosbuvir/ledipasvir had a resolution of viremia at the end of therapy that persisted 3 months later.

**Figure 2:**
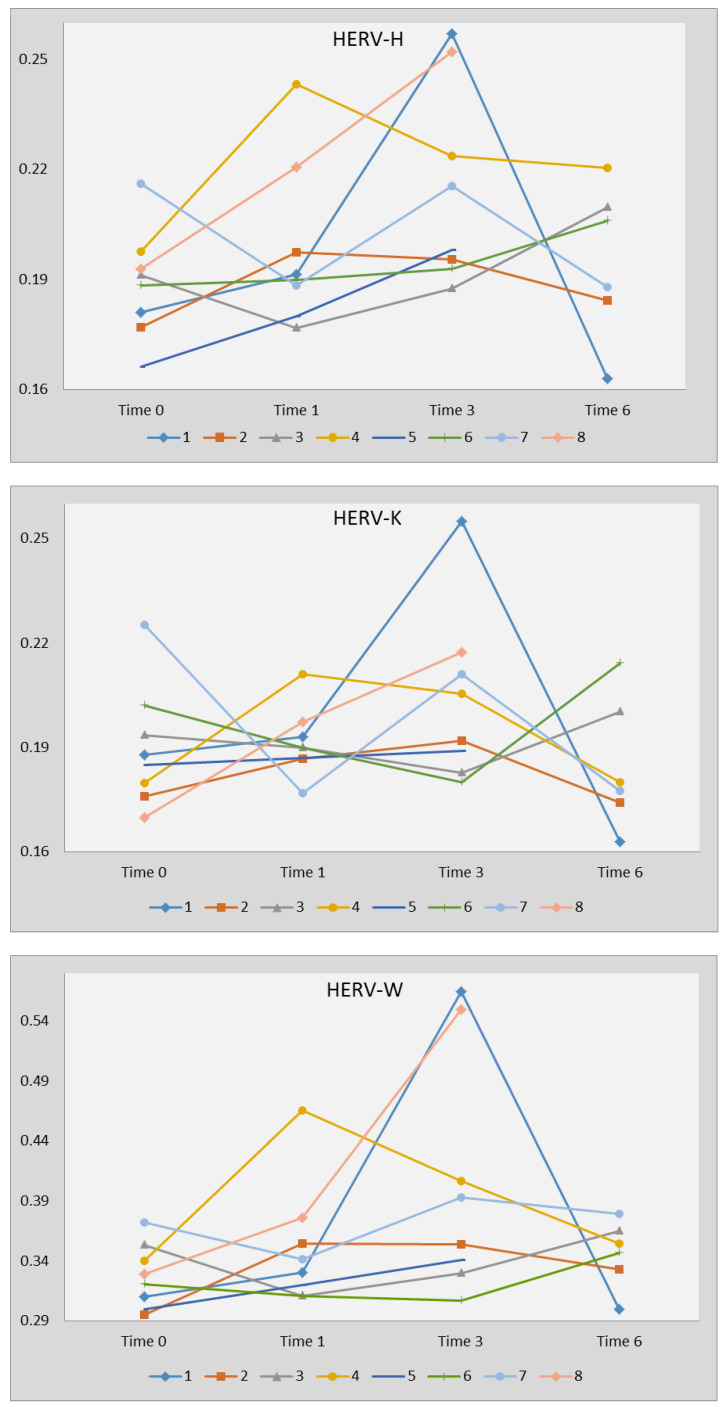
Transcription levels of the pol genes of HERV-H, HERV-K, and HERV-W in WBCs from HCV-infected subjects before (time 0), after 1 month (time 1) and at suspension (time 3) of sofosbuvir/ledipasvir therapy, and 3 months later. Transcription levels are represented by 1/ΔCt. Statistical analysis through two-way ANOVA test: HERV-H *p* = 0.1886, HERV-K *p* = 0.2884, and HERV-W *p* = 0.1619. Patients 1,3,4,5,7,8 were infected with genotype 1; patients 2 and 6 were infected with genotype 4.

Transcription levels of every HERV-pol gene did not change significantly before (time 0), after 1 month (time 1; 6/8 subjects tested) and at suspension (time 3) of sofosbuvir/ledipasvir therapy, and 3 months later (time 6; 6/8 subjects tested) (Figure 2).

In particular, the HERV-H-pol values (mean +/− SD) were 0.189 +/− 0.015 at time 0; 0.203 +/− 0.025 at time 1; 0.219 +/− 0.033 at time 3; 0.192 +/− 0.026 at time 6; HERV-K-pol values were: 0.190 +/− 0.017 at time 0; 0.193 +/− 0.011 at time 1; 0.207 +/− 0.030 at time 3; and 0.181 +/− 0.025 at time 6; and HERV-W-pol values were 0.326 +/− 0.027 at time 0; 0.363+/− 0.055 at time 1; 0.437 +/− 0.155 at time 3; and 0.342+/− 0.040 at time 6.

### 2.5. Expression Levels of TRIM28 in HCV RNA+ Subjects and Control Group

**Figure 3:**
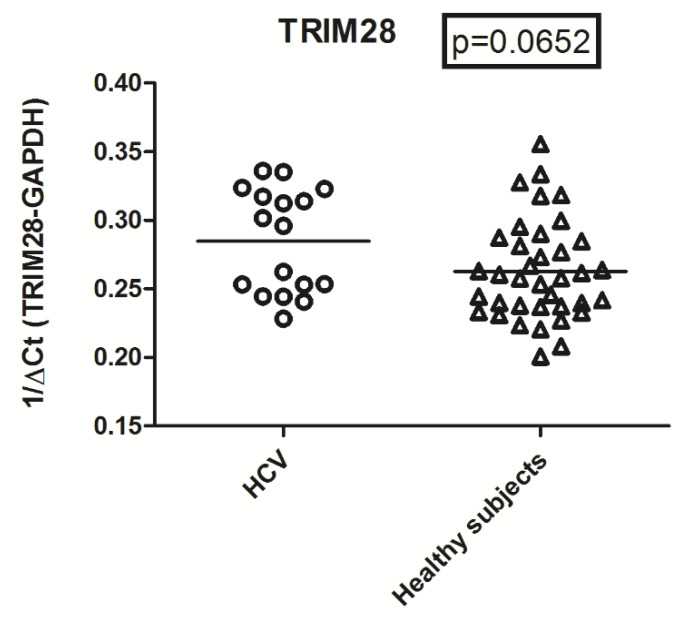
Transcription levels of TRIM28 in WBCs from HCV-infected subjects and age-matched control subjects. Circles and triangles show transcription levels of each subject; these are represented by 1/ΔCt. Statistical analysis through the Mann–Whitney test.

Expression levels of TRIM28 did not differ significantly between HCV RNA+ subjects and the control group (mean +/− SD: 0.289 +/− 0.035 vs. 0.263 +/− 0.036, respectively (Figure 3).

### 2.6. Expression Levels of TRIM28 Following Therapy with DAAs

**Figure 4:**
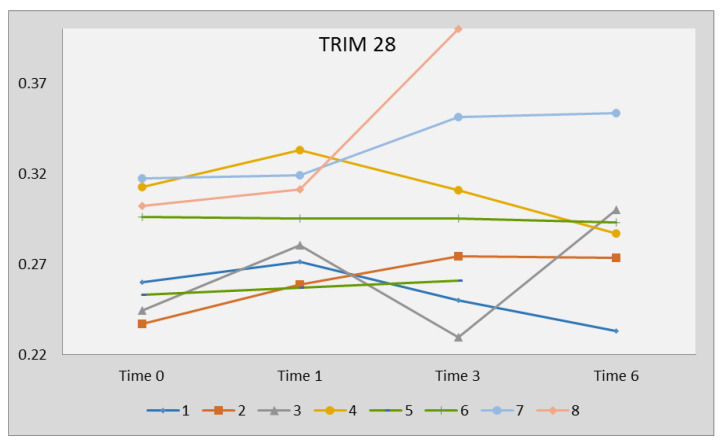
Transcription levels of TRIM28 in WBCs from HCV-infected adolescents before (time 0), after 1 month (time 1), and at suspension (time 3) of sofosbuvir/ledipasvir therapy, and 3 months later. Transcription levels are represented by 1/ΔCt. Statistical analysis through two-way ANOVA test: *p* = 0.7882.

No significant variations of TRIM28 were observed in subjects who underwent sofosbuvir/ledipasvir therapy: 0.278 +/− 0.032 at time 0, 0.296 +/− 0.030 at time 1, 0.316 +/− 0.086 at time 3, and 0.290 +/− 0.039 at time 6 (Figure 4).

## 3. Discussion

The results of our study show, for the first time, that HCV RNA+ children and adolescents with vertically acquired infection have significantly higher transcription levels of the pol genes of HERV-H and HERV-K in WBCs as compared to an age-matched control group. HERV-H-pol and HERV-K-pol overexpression remained unchanged during therapy and after DAA-induced viral clearance and was not attributable to reduced transcription of the TRIM28 repressor. In contrast, no dysregulation of HERV-W-pol RNA was observed between HCV-infected subjects and the control group.

The origin of enhanced HERV-H-pol and HERV-K-pol RNAs in subjects with chronic HCV infection remains to be elucidated. Several viral infections, such as herpesviruses [21,22,23], HIV [24] and influenza [25], can cause transactivation of retroviral sequences. Exogenous viral infections produce proinflammatory cytokines and type 1 interferons that lead to an independent and synergistic upregulation of endogenous retroviruses [26]. In particular, HCV infection triggers an inflammatory response via TLR/NF-kB pathway [27]. After nuclear translocation, the active isoform of NF-kB binds to specific sequences of HERV proviruses, and, with proinflammatory cytokines [26], it may give rise to the greater transcription of pol genes of HERV-H and -K found in our patients. In contrast, HERVW-pol sequences could be less responsive. HERV activation enhances the cell resistance to secondary infections through stimulation of innate immunity [28], but it exposes the cell to the potential negative impact of HERVs on the gene regulatory network and on physiological functions. In fact, endogenous retroviruses may enhance or suppress the transcription of adjacent cellular genes; through a copy and paste mechanism they may give rise to novel insertions into the DNA, and their RNAs, being sensed as nonself by PRR, can elicit inflammatory and immune reactions [12,29].

HERVs are tightly repressed transcriptionally by host TRIM28 [19,20]. This is an E3 ligase that mediates the transfer of ubiquitin or the small ubiquitin-like modifier (SUMO) to target substrates [30]. Among the virus-driven mechanisms responsible for HERV activation, it has been shown that influenza A virus elicits a SUMO-mediated metabolic switch with the modification status of TRIM28, resulting in the derepression of retroviral elements [25]. TRIM28 expression levels were higher, although without a statistically significant difference, in HCV-infected patients as compared to the control group and remained unchanged after DAA treatment. Therefore, the increased quantities of HERV-H-pol and HERV-K-pol RNAs observed in our patients cannot be ascribed to impaired transcription of TRIM28 repressor.

An intriguing aspect of our findings was that HERV-H-pol and HERV-K-pol transcripts did not change after DAA-induced viral clearance. This might be due to the fact that the HCV was not completely eliminated by treatment: an occult infection has actually been detected in WBCs from subjects with a positive response to DAA [31,32]. However, an indelible imprinting of HCV on activation of HERV sequences cannot be excluded.

Independently of the underlying mechanism(s), the upregulation of HERV-H and HERV-K in HCV-infected subjects might explain their high risk of cancers and autoimmune disorders [1,2,3,4,5] that persist after drug-driven viral clearance [6,7,10,11]. HERV-H is the most represented retroviral family in the human genome [33]. High levels of its fragments were detected in several cancer cells [16,34,35] representing a critical determinant of tumor progression and immune escape [36,37]. HERV-K is a recently acquired retroviral family, with formation into the human genome of specific sequences not present in other primates [38,39]. It has been widely studied and proposed as directly involved in carcinogenesis, tumor progression [39,40,41,42,43], and drug resistance [44]. HERV-K accessory proteins, such as Np9 and Rec, are putative potent oncogenes [38] and a few envelope proteins may facilitate cell transformation and metastasis [45]. It must be underlined that HERV-K upregulation has been found in patients with lymphoma [46] and HCC [47], typical tumors of HCV-infected subjects. Notably, the increase in HERV-K expression was significantly related not only to cancer evolution but also to development of cirrhosis [47]. On this basis, our results suggest that retroviral elements could also influence the HCV progression towards cirrhosis. The vicious circle: HCV-induced inflammation -> enhanced HERV transcription mediated by NF-kB/inflammatory cytokines -> HERV RNAs sensed as nonself by PRRs -> further inflammation may lead to progressive liver damage and reparatory fibrosis.

Besides its impact on cirrhosis and carcinogenesis, HERV activation may lead to alterations in the host immune system with autoimmune reactions like those observed in children with vertically acquired HCV infection [8,9]. An increasing number of clinical and experimental findings [17,18], including targeted research in animals [48,49,50], support the potential etiopathogenetic role of HERVs in triggering and/or maintaining autoimmune diseases [51]. Some retroviral epitopes can share molecular mimicry with components of body tissues or exogenous viruses. HERV-encoded proteins are targets of autoreactivity in patients with SLE [52]. Higher quantities of surface HERV-H env proteins have been found in leucocytes of patients with active multiple sclerosis, accompanied by increased specific seroreactivity [53]. A significant upregulation in HERV-K mRNA concentrations, [54] and elevated antibody response to a HERV-K-gag [55] and env [56] peptides, were found in subjects with rheumatoid arthritis, while a HERV-K superantigen was detected in those with juvenile arthritis [57]. Therefore, higher HERV expression in subjects with chronic HCV infection might justify their risk of developing malignancies and/or autoimmune disorders persisting after DAA-induced resolution of viremia. However, whether endogenous retroviruses really play a crucial pathogenetic role in these pathologies or just represent a secondary epiphenomenon remains questionable.

Some limits and doubts arise from our study. The primers and probes we used encompass all pol genes of HERV-H and HERV-K; therefore, no specific sequences could be identified and, thus, no possibility to associate a specific locus with a defined molecular pathogenetic mechanism. Furthermore, we did not assess their protein-coding capacity, even though HERVs, as noncoding regulatory elements, can induce or block the activity of neighboring cellular genes and their transcripts may be recognized as nonself by viral RNA receptors [29] with consequent alterations in the cellular homeostasis. Whereas a few children developed autoimmune phenomena, none had cirrhosis or cancers, which presumably require longer incubation periods. Consequently, the overexpression of HERVs found in patients with cirrhosis and HCC [47] needs to be confirmed in subjects with prolonged drug-driven viral clearance, to further support their putative role in these pathologies.

In conclusion, despite the small sample size, our study is the first to provide insights into the enhanced expression of some HERVs in HCV-infected subjects that does not fade after DAA-induced viral clearance. This durable activation of retroviral sequences might account for some HCV-related complications, such as the development of cancers and autoimmune diseases, that persist after the resolution of viremia. Targeted studies on larger study populations and sustained viral clearance are needed to shed further light on this intriguing aspect that can open new prophylactic and therapeutic strategies to avoid some complications linked to HCV infection.

## 4. Materials and Methods

### 4.1. Study Populations

HCV RNA+ children and adolescents with vertically acquired infection were studied. Of these, those older than 12 years underwent treatment with sofosbuvir/ledipasvir according to a national protocol [58]; the latter were tested before (time 0), after 1 month (time 1), and at suspension (time 3) of 3 months of therapy, and 3 months later (time 6).

Asymptomatic subjects of comparable age who were tested at the Regina Margherita Children’s Hospital, Turin, Italy, for routine laboratory examinations, and whose results were all within the normal reference range, were the control group. Subjects with any confirmed or suspected disease, such as infections, cancer, autoimmune disorders, inflammatory diseases, neurological disturbances, or abnormal laboratory results, were excluded from the study. The tests were performed using leftovers of laboratory samples; data were gathered anonymously.

### 4.2. Detection of HCV RNA

HCV RNA was detected by a real-time PCR assay (Cobas AmpliPrep/Cobas TaqMan HCV, Roche, Basel, Switzerland).

### 4.3. Total RNA Extraction and Retro-Transcription

Total RNA was extracted from WBCs using the automated extractor Maxwell (Promega, Madison, WI, USA) following the RNA Blood Kit protocol without modification. This kit provides treatment with DNase during the RNA extraction process. Four hundred nanograms of total RNA was reverse-transcribed with 2 μL of buffer 10X, 4.8 μL of MgCl_2_ 25 mM, 2 μL ImpromII (Promega), 1 μL of RNase inhibitor 20 U/L, 0.4 μL random hexamers 250 μM (Promega), 2 μL mix dNTPs 100 mM (Promega), and dd-water in a final volume of 20 μL. The reaction mix was carried out in a GeneAmp PCR system 9700 Thermal Cycle (Applied Biosystems, Foster City, CA, USA) under the following conditions: 5 min at 25 °C, 60 min at 42 °C, and 15 min at 70 °C for the inactivation of enzyme; the cDNAs were stored at −80 °C until use. For control of genomic DNA contamination, we amplified RNA extracts directly without reverse transcription.

### 4.4. Transcription Levels of Pol Genes of HERV-H, HERV-K, HERV-W and of TRIM28

Relative quantification of mRNA expression of HERV-H, -K -W and of TRIM28 was achieved by means of PCR real-time TaqMan amplification and normalization to glyceraldehyde-3-phosphate dehydrogenase (GAPDH) using the ABI PRISM 7500 real-time system (Life technologies, Carlsbad, CA, USA). GAPDH was chosen as the reference gene, being the most stable among 9 reference genes [59] and previously used in our studies [43,60,61,62]. A 40-ng quantity of cDNA was amplified using HERV-H, -K –W, and TRIM28 gene mRNA expression kit PP-BioMole-054, -055, -056, and 044 respectively (BioMole srl, Turin, Italy) in a 20 μL total volume reaction.

The amplifications were run in a 96-well plate at 95 °C for 10 min, followed by 40 cycles at 95 °C for 15 s, and at 60 °C for 1 min. Furthermore, in order to confirm that there was no DNA genomic contamination, control PCR was performed with RNA before reverse transcription, using the same primers and probes described above. Each sample was run in triplicate. Relative quantification of target gene expression was performed with the ΔCt method. Using 40 ng of cDNA in amplification, we obtained Ct values from 26 to 30.4. These Ct values correspond to a good performance of real-time PCR. Since we measured Ct for every target in all the samples tested, we argued that our methods were suitable for HERV detection and quantification. Results were expressed as 1/ΔCt.

### 4.5. Statistical Analysis

The Mann–Whitney test was used to compare the transcriptional levels of pol genes of every HERV family as well as of TRIM28 in WBCs from HCV-infected subjects vs. age-matched controls. A two-way ANOVA test was used to compare the transcriptional levels of each HERV group before (time 0), after 1 month (time 1), and at suspension (time 3) of sofosbuvir/ledipasvir therapy, and 3 months later (time 6). Statistical analyses were done using the Prism software (GraphPad Software, La Jolla, CA, USA). In all analyses, *p* < 0.05 was taken to be statistically significant.

Clinical data were treated in accordance with the principles of Helsinki Declaration (World Medical Association General Assembly, Seoul, Korea, October 2008). The study protocol was approved by AIFA (24.04.2018) and Azienda Ospedaliera-Universitaria Città della Salute e della Scienza, Turin (final amendment 20.04.2020); code 289571.

## Abbreviations

ANA	Anti-nuclear antibodies
DAAs	Direct-acting antivirals
GAPHD	Glyceraldehyde-3-phosphate dehydrogenase
HCC	Hepatocellular carcinoma
HCV	Hepatis C virus
HERVs	Human endogenous retroviruses
LKM-1	Liver and kidney microsomal type 1
NF-kB	Nuclear factor kB
PCR	Polymerase chain reaction
PRRs	Pattern recognition receptors
SLE	Systemic lupus erythematous
TLR	Toll-like receptor
TRIM28	Tripartite motif-containing-28
WBCs	White blood cells
